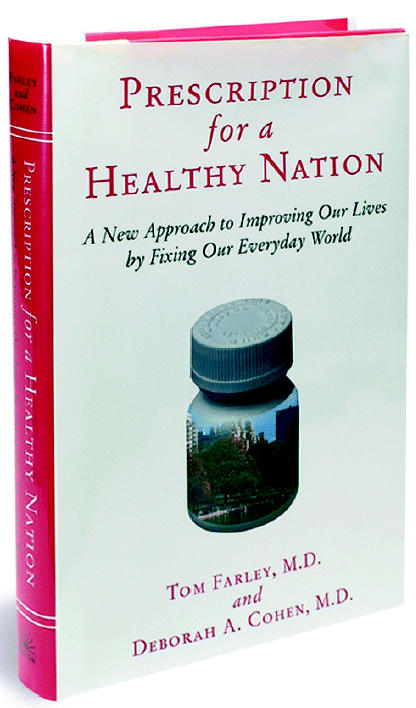# Prescription for a Healthy Nation: A New Approach to Improving Our Lives by Fixing Our Everyday World

**Published:** 2005-09

**Authors:** Rudolph Rull

**Affiliations:** Rudolph Rull is an assistant researcher at the Center for Health Policy Research at the University of California, Los Angeles. He is an epidemiologist whose research focuses on using geographic information systems for assessing environmental exposures to pesticides and air pollution and characterizing the built environment.

By Tom Farley and Deborah Cohen

Boston, MA:Beacon Press, 2005. 243 pp. ISBN: 0-8070-2116-4, $24.95 cloth

Much of our daily news is devoted to health care crises. Spiraling health care costs, which currently consume one-seventh of the U.S. economy, are mostly devoted to treating a modern epidemic of preventable chronic conditions such as obesity, diabetes, lung cancer, and heart disease; however, treatment of these chronic conditions does little to prevent future cases. Preventive interventions designed to educate people about the health risks of overeating, tobacco use, alcohol abuse, and sedentary lifestyles have had limited success in modifying behaviors and have not translated into a logical reversal in the prevalence of these chronic diseases.

In *Prescription for a Healthy Nation*, Tom Farley and Deborah Cohen suggest solutions for reversing this trend and preventing new cases by changing the social and physical environment. In “The Leading Causes of Health,” the authors argue that the environment is a powerful force in influencing human behavior, although changing and redesigning the environment to promote health is not necessarily a “new approach.” The sanitation and hygiene revolution of the 19th and 20th centuries—the greatest success of modern public health practice—was largely carried out by constructing sewers, mandating solid waste disposal and access to clean water in cities, and other interventions to prevent the spread of infectious agents. The success of these preventive measures lies in the fact that the benefits affect the entire society. However, according to the authors, many current preventive interventions that attempt to reduce risk through education tend to ignore the aspects of our environment that encourage risky behavior, such as the overabundance of high-fat junk food and pervasive advertising of alcohol and tobacco. In addition, preventive education has largely targeted individuals at high risk such as people with high-fat diets who are thus at a higher risk for having a heart attack, while neglecting the much larger population of individuals with moderate- or low-fat diets who have a lower risk for heart attack. By absolute numbers, more lower-risk people will suffer heart attacks than high-risk individuals; therefore, consuming less fat will reduce everyone’s risk. The authors propose a return to this “curve-shifting” approach to prevention that encourages everyone to improve their health behavior; they substantiate these arguments with examples from public health and cognitive psychology research, historical and current events, and personal anecdotes.

In “Curve Shifters,” Farley and Cohen identify four modifiable components that influence our health environment or “healthscape”: *a*) accessibility of healthy (e.g., fruits and vegetables) and unhealthy items (e.g., tobacco); *b*) physical structures that promote or endanger health (e.g., guardrails) and neighborhood designs that discourage crime or promote physical activity; *c*) social structures that influence the acceptability of our health behaviors (e.g., bans on indoor smoking); and *d*) the popular media that influences our behavior through advertising and the broadcasting of influential images in movies.

In the final section, “Healthscaping America,” solutions are proposed for altering the environment to promote health, for example, mandating the display of fresh fruits and vegetables at checkout counters in supermarkets and convenience stores and banning the sale of junk food in schools and the advertising of alcohol on television. Workplaces can encourage employees to take exercise breaks, and neighborhood streets can be designed to encourage walking and bicycling for travel and recreation. The authors concede that these are not complete solutions and that these proposals will generate controversy and be viewed both as radical and as an excuse for personal irresponsibility by policymakers and businesses with a financial stake in their implementation. However, throughout the history of public health, interventions such as sanitation and indoor smoking bans that were once deemed radical are now commonly accepted as a responsibility of the state.

Farley and Cohen present these provocative ideas in a clear and highly readable manner with contemporary examples that address the urgency of this crisis. This book is an instructive resource for scientists, policymakers, community health advocates, and anyone with an interest in improving the health of our society.

## Figures and Tables

**Figure:**